# Adjustment Effects of Maximum Intensity Tolerance During Whole-Body Electromyostimulation Training

**DOI:** 10.3389/fphys.2019.00920

**Published:** 2019-07-24

**Authors:** Joshua Berger, Stephan Becker, Marco Backfisch, Christoph Eifler, Wolfgang Kemmler, Michael Fröhlich

**Affiliations:** ^1^Department of Sports Science, Technische Universität Kaiserslautern, Kaiserslautern, Germany; ^2^Department of Applied Training Science, German University of Applied Sciences for Prevention and Health Management (DHfPG), Saarbrücken, Germany; ^3^Institute of Medical Physics, Friedrich Alexander University of Erlangen-Nürnberg, Erlangen, Germany

**Keywords:** adjustment effects, familiarization, intensity tolerance plateau, specific adaptations, whole-body electromyostimulation

## Abstract

Intensity regulation during whole-body electromyostimulation (WB-EMS) training is mostly controlled by subjective scales such as CR-10 Borg scale. To determine objective training intensities derived from a maximum as it is used in conventional strength training using the one-repetition-maximum (1-RM), a comparable maximum in WB-EMS is necessary. Therefore, the aim of this study was to examine, if there is an individual maximum intensity tolerance plateau after multiple consecutive EMS application sessions. A total of 52 subjects (24.1 ± 3.2 years; 76.8 ± 11.1 kg; 1.77 ± 0.09 m) participated in the longitudinal, observational study (38 males, 14 females). Each participant carried out four consecutive maximal EMS applications (T1–T4) separated by 1 week. All muscle groups were stimulated successively until their individual maximum and combined to a whole-body stimulation index to carry out a possible statement for the development of the maximum intensity tolerance of the whole body. There was a significant main effect between the measurement times for all participants (*p* < 0.001; 𝜂^2^ = 0.39) as well as gender specific for males (*p* = 0.001; 𝜂^2^ = 0.18) and females (*p* < 0.001; 𝜂^2^ = 0.57). There were no interaction effects of gender × measurement time (*p* = 0.394). The maximum intensity tolerance increased significantly from T1 to T2 (*p* = 0.001) and T2 to T3 (*p* < 0.001). There was no significant difference between T3 and T4 (*p* = 1.0). These results indicate that there is an adjustment of the individual maximum intensity tolerance to a WB-EMS training after three consecutive tests. Therefore, there is a need of several habituation units comparable to the identification of the individual 1-RM in conventional strength training. Further research should focus on an objective intensity-specific regulation of the WB-EMS based on the individual maximum intensity tolerance to characterize different training areas and therefore generate specific adaptations to a WB-EMS training compared to conventional strength training methods.

## Introduction

Electromyostimulation training (EMS training) has been used since the early 1970s as a high-intensity training technology in high-performance sports, physical therapy, and rehabilitation (Selkowitz, 1985, 1989; Duchateau and Hainaut, 1988; Binder-MacLeod and McDermond, 1992; Filipovic et al., 2011, 2012; Kemmler et al., 2018). Electrodes attached to the skin lead to involuntary contraction of the muscles underneath the electrodes while in conventional strength training, voluntary muscle contraction against a resistance takes place. In conventional strength training, differentiated intensities are derived to control training and generate specific adaptations of the musculature to athletic exercise. These intensities are often based on the one-repetition-maximum (1-RM). The 1-RM has been established as a simple, economic, and adequately valid option to determine training intensity levels. It is considered the gold standard of muscle strength evaluation in non-laboratory conditions (LeSuer et al., 1997; Ritti-Dias et al., 2005; Levinger et al., 2009; Kenney et al., 2015). Due to the complexity of movement and neuronal adaptation mechanisms, strength training beginners are often not able to exploit their absolute maximum capabilities the first time they exercise. Only after repeated units, the personal best may be reached at all (Rutherford and Jones, 1986; Mayhew et al., 1989; Braith et al., 1993; Ritti-Dias et al., 2005, 2011; Wirth et al., 2012). This means that athletes require consecutive stimuli until they are able to reach a certain plateau or their individual maximum. This individual learning and familiarization phase is said to be unexplored in EMS training. Furthermore, there is insufficient knowledge in the fields of deriving percentage training intensities and determining individual maximum intensity tolerances or an equivalent to the 1-RM.

The individual training fields for EMS training are usually specified by assessing the degree of perceived exertion based on the Borg RPE (rating of perceived exertion) scale (Borg, 1998; Kemmler et al., 2012, 2016b,c,d; Kemmler and von Stengel, 2012; Amaro-Gahete et al., 2018). To date, objectified training intensities have not been measured. In their work, Alon and Smith already examined the adaptation to an EMS application after several consecutive sessions and found that the maximum intensity tolerance continuously increases (Alon and Smith, 2005). However, they focused on one muscle only (m. quadriceps femoris) and the combination of an EMS application and maximum voluntary isometric contraction (MVC). Familiarization and adaptation effects regarding a whole-body EMS training have not been identified yet. Training guidelines recommend an 8- to 10-week adaptation phase in order to avoid unwanted metabolic effects. This, however, does not allow any statement on the individual maximum intensity tolerance (Kemmler et al., 2016a).

Therefore, the aim of this study is to determine whether a maximum intensity tolerance plateau occurs after multiple consecutive whole-body electromyostimulation (WB-EMS) application sessions.

## Materials and Methods

### Study Design and Subjects

A total of 59 test subjects participated in the longitudinal, observational analysis in a panel design. Four consecutive tests were performed at the same time of day with a 1-week interval. Due to health complications and further personal reasons (not related to the WB-EMS application), seven persons did not perform four consecutive tests, so that in the end, 38 male and 14 female participants (*n* = 52) were entered in the study. Participants’ characteristics are shown in Table 1.

**Table 1:** Anthropometric data for the entire group (*n* = 52) and the gender subgroups, shown in mean value ± standard deviation; BMI = body mass index.

	*N*	Height [cm]	Weight [kg]	Age [years]	Body fat [%]	BMI [kg/m^2^]
General	52	177.9 ± 8.9	76.76 ± 11.06	24.13 ± 3.28	18.14 ± 7.67	24.20 ± 2.31
Women	14	167.8 ± 5.1	65.84 ± 7.94	23.84 ± 3.00	28.29 ± 4.48	23.35 ± 2.28
Men	38	181.6 ± 6.6	80.79 ± 9.21	24.93 ± 3.95	14.40 ± 4.56	24.52 ± 2.27

All participants were EMS beginners, recruited via e-mail distribution lists, flyers, and personal contact. Exclusion criteria were acute or chronic diseases, infections or limitations to the musculoskeletal system, EMS experience in the past, and open skin lesions, which would have inhibited EMS application. The subjects had to be between 18 and 40 years of age with a BMI < 30 kg/m^2^, and they were not allowed to take any medication that might have affected the examination (pain medication, beta blockers, etc.). Before study start, a detailed anamnesis questionnaire had to be completed in order to exclude any relative and/or absolute contraindications for EMS training (Vatter et al., 2016; Kemmler et al., 2016a). Furthermore, the course of the study, the testing design, and potential risks were explained in detail. Before entering the study, all participants provided written informed consent to the experimental procedure and written consent pertaining to data use. The study was conducted based on the current Declaration of Helsinki guidelines (World Medical Association, 2013) and approved by the ethics commission of the German University of Applied Sciences for Prevention and Health Management (project number 02/17).

### Materials

**Figure 1:**
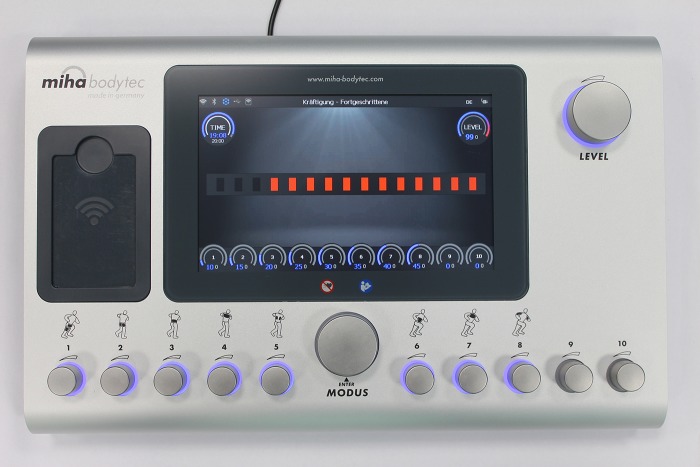
WB-EMS device miha bodytec 2.

The WB-EMS application was carried out with the miha bodytec 2 EMS device from Miha Bodytec. The device includes one main controller to manage the possible maximal output of the device and 10 subcontrollers to dose the specific muscle groups (Figure 1). In this investigation, we just used the eight prepared muscle groups with no additional muscle groups using the canals 9 and 10. The muscle groups were stimulated using an electrode vest including five pairs of electrodes (lower back, latissimus, upper back, abdomen, and chest), a hip belt to stimulate the glutaeus muscle and one belt pair each for the thighs and the upper arms. To avoid the direct contact of the electrodes to the skin, a special functional EMS lingerie was used. All the electrodes (vest and belts) were moistened before use to guarantee a better conductivity from the electrodes to the participants’ body. The EMS application and the way of using the workout clothes were in line with the manufacturer’s instructions (Miha Bodytec, Augsburg, Germany). Body fat was determined using the Tanita BC-418 body composition analyzer with the software GMON v.3.2.3. 10. The participants were instructed to drink 1 litre of water up to 60 min before the bioimpedance analysis to reduce the variability of the results.

### Tests

The tests were conducted based on common EMS application stimulation parameters, i.e., a frequency of 85 Hz, an impulse width of 350 μs, and a bipolar impulse without impulse increase (rectangular impulse) with interchanging 4-s load and 4-s break intervals (Filipovic et al., 2011; Kemmler et al., 2012, 2018).

Each muscle group was strained in intervals up to their individual, subjective maximum. The maximum was determined by the point at which the test person gave the signal to stop the strain due to the highest degree of strain that could be endured. The last stimulation value output by the EMS device was therefore recorded as the maximum intensity tolerance. This is, however, just a numerical value that cannot be equated with a specific value in milliamperes (mA). All maximum intensity tolerances refer to the stimulation of a specific muscle. The device was set up so that a maximum output and thus maximum device load were possible [maximal value of the main level controller (99)]. All muscle groups were strained consecutively in this way. During the tests, the test persons were never informed about the stimulation value or the maximum intensity tolerances of any of the previous tests. This resulted in a maximum intensity tolerance range of 0–99 (device-specific unit, 0–75 mA) for each muscle group. For a better understanding, Figure 1 shows the miha bodytec 2 device including the main level controller as well as the specific muscle groups. The individual muscle groups were combined into an unweighted, additive index in order to come to a statement on the development of the entire body and not only a specific muscle group. After a preceding impulse adaptation, each test person took part in four consecutive tests in 1-week intervals. Four tests were selected for better comparability with the approach to the determination of 1-RM in conventional strength training. A separation of 1 week was used to guarantee complete regeneration after each session. In order to exclude time-of-day effects, the tests were performed at the same time of day for each test person. In addition, the participants were always examined by the identical research staff. Before each test, a new anamnesis questionnaire on the current condition was completed in order to exclude spontaneously occurring contraindications (intake of pain medication or alcohol, muscle aches, liquid receptive before the training, etc.).

### Statistics

Sample size was calculated using G*power 3.1.9.4 (university of Düsseldorf, Germany). With an effect size of 0.25, total sample size was calculated by 36 participants (Faul et al., 2007). With regard to possible drop-outs, more participants were admitted to the study. Statistical evaluation and graphics generation were executed using IBM SPSS (SPSS Version 25.0, Chicago, IL, USA). The normal distribution was verified by means of the Shapiro-Wilk test. Because of this criteria, to check the maximum intensity tolerance index development, the four test times T1–T4 were analyzed by means of repeated-measures analysis of variance. Since ANOVA is known to be robust when it comes to infringements of the normal distribution, it was applied every time (Schmider et al., 2010). *Post hoc* comparisons including Bonferroni correction were performed to detect differences between the measurement times. As the Mauchly test indicated a violation of sphericity, the degrees of freedom corrected according to Huynh-Feldt were used for the further calculation of *p* [because epsilon by Greenhouse Geiser >0.75 (Girden, 1992; Field, 2009)]. Furthermore, the effect size *𝜂*^2^ was calculated (Cohen, 1988). The significance level was set to *p* < 0.05.

## Results

Since the device-specific maximum was reached for eight test persons during the four test dates, the number of valid data records was reduced to 44. Table 2 represents the index values of the individual test days. Between the measurement times, a significant main effect *F* (2.46; 105.96) = 26.95, *p* < 0.001, *𝜂*^2^ = 0.39 was identified, which categorizes the effect size as large according to Cohen. To differentiate between genders, the degrees of freedom corrected according to Greenhouse Geiser were applied. For the male test persons, a significant main effect was identified *F* (2.21; 77.41) = 7.66, *p* = 0.001, *𝜂*^2^ = 0.18. Also for the female test persons, a significant main effect was found between the measurement times with *F* (1.84; 22.07) = 15.82, *p* < 0.001, *𝜂*^2^ = 0.57. Significant main effects were also identified pertaining to the gender factor: [*F* (1; 45) = 12.09, *p* = 0.001, *𝜂*^2^ = 0.21]. There were no significant interaction effects for gender multiplied by measurement time [*F* (3; 135) =1.00, *p* = 0.394, *𝜂*^2^ = 0.02].

**Table 2:** Maximum intensity tolerance as mean value ± standard deviation and 95% confidence interval [CI] of the measuring times T1–T4 for the entire collective and men and women.

Time of measuring	Maximum intensity tolerance of all participants [CI]	Maximum intensity tolerance of men [CI]	Maximum intensity tolerance of women [CI]
T1	61.03 ± 11.71 [57.59–64.47]	64.67 ± 10.15 [61.13–68.22]	51.49 ± 10.31 [45.25–57.72]
T2	64.57 ± 10.50 [61.48–67.65]	67.02 ± 9.62 [63.65–70.37]	58.18 ± 10.35 [51.92–64.42]
T3	68.97 ± 10.97 [65.75–72.19]	71.25 ± 9.42 [67.96–74.54]	63.03 ± 12.83 [55.27–70.78]
T4	70.46 ± 13.62 [66.46–74.46]	73.50 ± 13.03 [68.95–78.04]	62.51 ± 12.26 [55.10–69.92]

**Figure 2:**
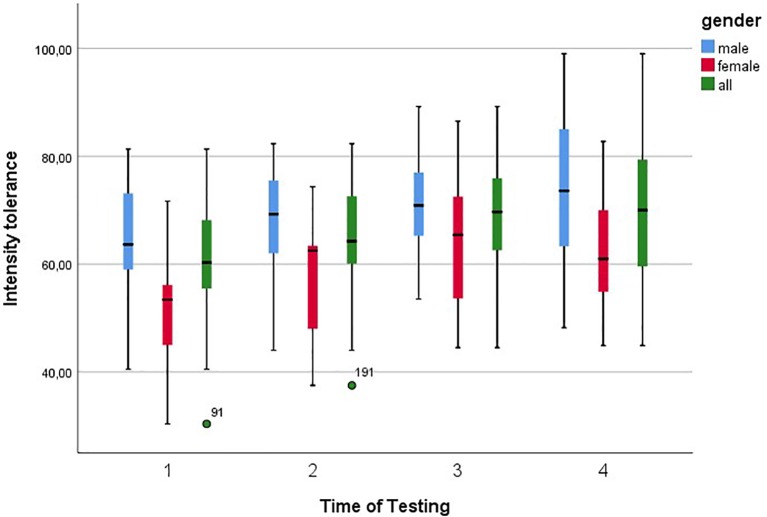
Boxplots of intensity tolerance with 95% confidence interval for all four times of testing.

There were significant differences between the measurement times T1 to T2 and T2 to T3. There were no significant differences between T3 and T4. The maximum intensity tolerance index values for the various measurement times (including confidence interval for men and women) as well as the percentage increases over the measurement times are included in Tables 2 and 3. For improved understanding, the boxplots for all test person data and gender specifics are shown in Figure 2.

**Table 3:** Comparison of pairs of testing sessions including significance and increase in %.

Comparison of pairs	*p*	Increase (%)
T1–T2	0.001	6.4
T2–T3	0.000	8.1
T3–T4	1.000	0.4

## Discussion

The results of this study show that a plateau develops after the third test session of consecutive measurements of the maximum intensity tolerance. A possible reason could be the habituation to the external electrical stimulation on the one hand and the muscular-coordinative adaptations on the intramuscular level on the other hand. Also neuronal adaptation caused by the maximal stimulation could be important. Therefore, the maximum intensity tolerance should be determined based on several consecutive test units in order to exclude the named influencing factors in the interpretation of results. This approach seems to be similar to that pertaining to the determination of the 1-RM, for which a certain number of familiarization and adaptation sessions is required to reach the individual maximum (Reynolds et al., 2006; Ritti-Dias et al., 2011). In conventional strength training, usually 2 weeks of learning and adaptation training with four measurement sessions precede the actual 1-RM determination so that the training intensity can be derived based on the 1-RM. Similarly, the determination of the individual maximum intensity tolerance through preceding adaptation sessions could serve as a preparation for the whole-body EMS training (Ritti-Dias et al., 2011). So far, one habituation session to an EMS training is recommended, which seems not sufficient to account all habituation and learning effects to a maximaum electrical intensity.

Previous analyses did not identify any intensity tolerance adaption in consecutive tests. For example, Alon and Smith (2005) conducted six NMES (neuromuscular electrical stimulation) sessions with 21 test persons within 2 weeks to see whether an adaptation of the maximum intensity tolerance would occur. During these examinations, they did not identify any adaptations. This could have been due to the isolated stimulation of the right-hand side m. quadriceps femoris in contrast to our whole-body index of maximum intensity tolerance (Alon and Smith, 2005). It remains to be clarified with more test persons whether there is a difference between beginners and experienced subjects at the point of plateau like in the 1-RM bench pressing and squats tests by Ritti-Dias et al. (2011). First studies by Hortobágyi et al. (1992) with 12 test persons point to a difference in maximum intensity tolerance between trained and untrained subjects. The trained persons exhibited a strength value for the m. biceps brachii that was up to 29% higher than that of the untrained participants. With 31.3 mA measured for the trained and 21.9 mA for the untrained subjects, the tolerated intensity strength was clearly higher in the trained subjects (Hortobágyi et al., 1992). The difference of the sensory threshold between males and females, i.e., the response to an external impulse, also seems to have been confirmed in previous tests. Maffiuletti et al. (2008) analyzed this with the help of 20 men and 20 women. They identified gender differences at the sensory threshold: the values of the female test persons were 41% lower than those of the male test persons (Maffiuletti et al., 2008). This is a result that we were able to corroborate based on the gender-specific differences in the maximum intensity tolerance (men were 13% higher than women). The results by Alon and Smith are thus confirmed. They found a significantly higher intensity tolerance in males (31.5 ± 8.6 μC) than in females (16.9 ± 8.0 μC). However, the percentage increase between the first and last unit conducted turned out to be mostly identical (male: 47.2%; female: 50.8%) (Alon and Smith, 2005). Similar gender-specific differences were observed by Fehr in pilot studies, with a difference of the maximum intensity tolerance of 35.9 mA in males and 22.3 mA in females (Fehr, 2011). These studies support our results because the intensity tolerance when developing the plateau is also higher in males in our tests. The course beyond the testing times, however, does not seem to be subject to any gender-specific differentiation.

In order to be able to perform the training studies with the appropriate intensity in the future and to derive conclusions from conventional strength training for EMS, it may be useful to specify a 1-RM equivalent to establish objective intensity ranges. The key question as to the extent to which percentage values of the individual intensity tolerances actually represent individual training areas such as maximum strength training, muscle hypertrophy, or rather strength-endurance training also remains to be clarified in future studies. Since strain intensity (i.e., the intensity in WB-EMS training) is just a control parameter for training adaptations, the methodology to be applied to WB-EMS training itself still needs to be defined. Due to a methodology’s essential influence on intensity perception and perceived exertion, it may actually represent a key factor in the establishment of training intensity based on its individual maximum.

## Conclusion

In summary, after multiple consecutive EMS application sessions with EMS beginners, a maximum intensity tolerance plateau was reached after three adaptation sessions. Therefore, future studies should plan for at least three adaptation sessions preceding the actual whole-body EMS training. We recommend that follow-up studies take the maximum intensity tolerance findings as a basis for defining percentage training areas in order to enable objective training control in the future. It is also recommended that the increase of the individual maximum intensity tolerance over a prolonged period of time and its potential changes caused by training adaptation should be verified. Also at a sub-maximum level, training intensity development should be observed in order to be able to derive relevant conclusions. Even if intensity is only one of several measures of training control in EMS training, it could be a key value to define training intensity, similar to 1-RM.

## Ethics Statement

This study was carried out in accordance with the recommendations of the Declaration of Helsinki with written informed consent from all subjects. The protocol was approved by the ethics commission of the German University of Applied Sciences for Prevention and Health Management (project number 02/17).

## Author Contributions

JB, SB, and MB participated in the design of the study, carried out the experiments, and performed data analyses. JB wrote the manuscript. MF and SB helped write the manuscript and supervised statistical analysis. MF, CE, and WK contributed to the study design and supervised the entire project. All authors read and approved the final manuscript.

### Conflict of Interest Statement

The authors declare that the research was conducted in the absence of any commercial or financial relationships that could be construed as a potential conflict of interest.
